# Pulmonary Alveolar Proteinosis-Like Presentation of Murine Typhus in a Previously Healthy Adult

**DOI:** 10.7759/cureus.114577

**Published:** 2026-08-15

**Authors:** Madeline Pan, Patrick Sweet

**Affiliations:** 1 John Sealy School of Medicine, University of Texas Medical Branch at Galveston, Galveston, USA; 2 Internal Medicine, University of Texas Medical Branch at Galveston, Galveston, USA

**Keywords:** crazy paving, ground-glass opacities, murine typhus, pulmonary alveolar proteinosis, rickettsia typhi

## Abstract

*Rickettsia typhi*-induced murine typhus is underrecognized in southern U.S. regions such as Texas. While typical symptoms include fever, rash, and mild pulmonary involvement, severe presentations such as pneumonia or acute respiratory distress syndrome (ARDS) can occur. The radiographic “crazy-paving” pattern is nonspecific and can be seen in a variety of infectious and inflammatory pulmonary conditions, creating diagnostic uncertainty. Because pulmonary alveolar proteinosis (PAP) typically prompts consideration of invasive diagnostic evaluation, recognizing infectious mimics has important clinical implications.

A 46-year-old man with alcohol use disorder develops fatigue, cough, dysuria, dyspnea, and fever. CT imaging revealed bilateral perihilar ground-glass opacities with interlobular septal thickening (“crazy-paving”). Laboratory studies showed leukocytosis, thrombocytopenia, acute kidney injury, transaminitis, and elevated inflammatory markers. Broad-spectrum antibiotics did not provide symptom relief. Serologic testing confirmed murine typhus; doxycycline led to rapid clinical and radiographic improvement. Four-week follow-up imaging showed near-complete resolution.

This is, to our knowledge, the first reported case of murine typhus presenting with radiological and clinical features that closely mimic pulmonary alveolar proteinosis. In endemic regions, murine typhus should be included in the differential diagnosis of diffuse alveolar disease with a crazy-paving pattern, as this overlap may lead to diagnostic uncertainty and potentially unnecessary invasive evaluation. Early recognition and treatment with doxycycline can result in rapid clinical and radiographic resolution.

## Introduction

Murine typhus remains endemic to coastal subtropical and tropical regions, such as in the southern U.S. states. The causative organism is *Rickettsia typhi *(*R. typhi*), a gram-negative bacillus, and the vector for the disease in humans can be rat or cat fleas. The pathophysiology of the disease is characterized by *Rickettsia*-mediated endothelial cell injury, leading to increased vascular permeability, extravasation of intravascular fluid, and subsequent multisystem organ involvement. This mechanism of disease contributes to the varying clinical presentation of typhus. Symptoms can include fever, headache, rash, thrombocytopenia, and elevated hepatic enzymes [1]. Pulmonary involvement occurs in approximately 30% of cases and may range from mild infiltrates to acute respiratory distress syndrome (ARDS) [2].

Pulmonary alveolar proteinosis (PAP) is a rare disorder characterized by alveolar filling with surfactant due to dysfunctional or depleted alveolar macrophages. Secondary PAP (sPAP) may be triggered by infections, hematologic malignancy, immunodeficiency, or environmental exposures [3]. The alveolar macrophage plays a critical role in surfactant clearance, and its dysfunction, whether immune-mediated or secondary to environmental or infectious insult, disrupts this balance [4]. The “crazy‑paving” CT pattern of ground‑glass opacities with interlobular septal thickening is classically associated with PAP but is nonspecific and may be seen in a range of infectious and inflammatory processes, including pulmonary edema, diffuse alveolar hemorrhage, Pneumocystis jirovecii pneumonia, organizing pneumonia, and ARDS [5,6]. Because these entities can present with overlapping radiographic and clinical findings, distinguishing PAP from its mimics may be diagnostically challenging, particularly early in the disease course.

## Case presentation

A 46-year-old man with a past medical history of alcohol use disorder presented with complaints of fatigue, cough, dysuria, dyspnea, and fever that started during the past week. The patient reported that he had taken acetaminophen and ibuprofen three to four times a day for his fever without avail and noticed increased distention in the abdomen despite decreased food intake this past week. His last consumption of alcohol was three pints of beer five days ago, and the patient denies daily drinking. He also mentioned having a rash that resolved on its own several days ago. He has performed maintenance ventilation work in a building with old air conditioning units for the past couple of weeks, which is something he has not done in a long time. Patient denies any other exposures and denies smoking, vaping, or any drug use. Vitals upon admission were significant for hypoxemia and mildly low blood pressure (Table 1).

**Table 1:** Patient's vitals upon admission indicating hypoxemia SpO_2_: oxygen saturation

Vital sign	Measurement
Blood pressure	98/69
SpO_2_	89% on room air
Pulse	91
Temperature	36.4 °C (97.6 °F)
Respiratory rate	18

Physical exam was notable for abdominal distension and fine crackles in the lower left lung. Laboratory studies showed leukocytosis, thrombocytopenia, acute kidney injury, transaminitis, and elevated procalcitonin (Table 2). In order to improve the patient's oxygen saturation, he was started on a 5-liter nasal cannula.

**Table 2:** Laboratory findings Patient's laboratory studies suggest an infection due to elevated white blood count, elevated procalcitonin, and thrombocytopenia. Of further note, the patient also presented with signs of acute kidney injury, liver injury, and electrolyte abnormalities. ALT: alanine aminotransferase; AST: aspartate aminotransferase; Hgb: hemoglobin; Na: sodium; WBC: white blood cell count

Laboratory parameters	Patient's values	Reference range
WBC	12.72 x 10^3/μL	4.20-10.70 x 10^3/μL
Hgb	12.1 g/dL	13.5-17.5 g/dL
Platelets	45 x 10^3/μL	150,000-400,000 x 10^3/μL
Na	128 mmol/L	136-146 mmol/L
Creatinine	1.83 mg/dL	0.6-1.2 mg/dL
Total bilirubin	1.8 mg/dL	0.1-1.0 mg/dL
Calcium	6.9 mg/dL	8.4-10.2 mg/dL
Procalcitonin	9.7 ng/mL	<0.07 ng/mL
ALT	234 U/L	10-40 U/L
AST	378 U/L	12-38 U/L

**Figure 1:**
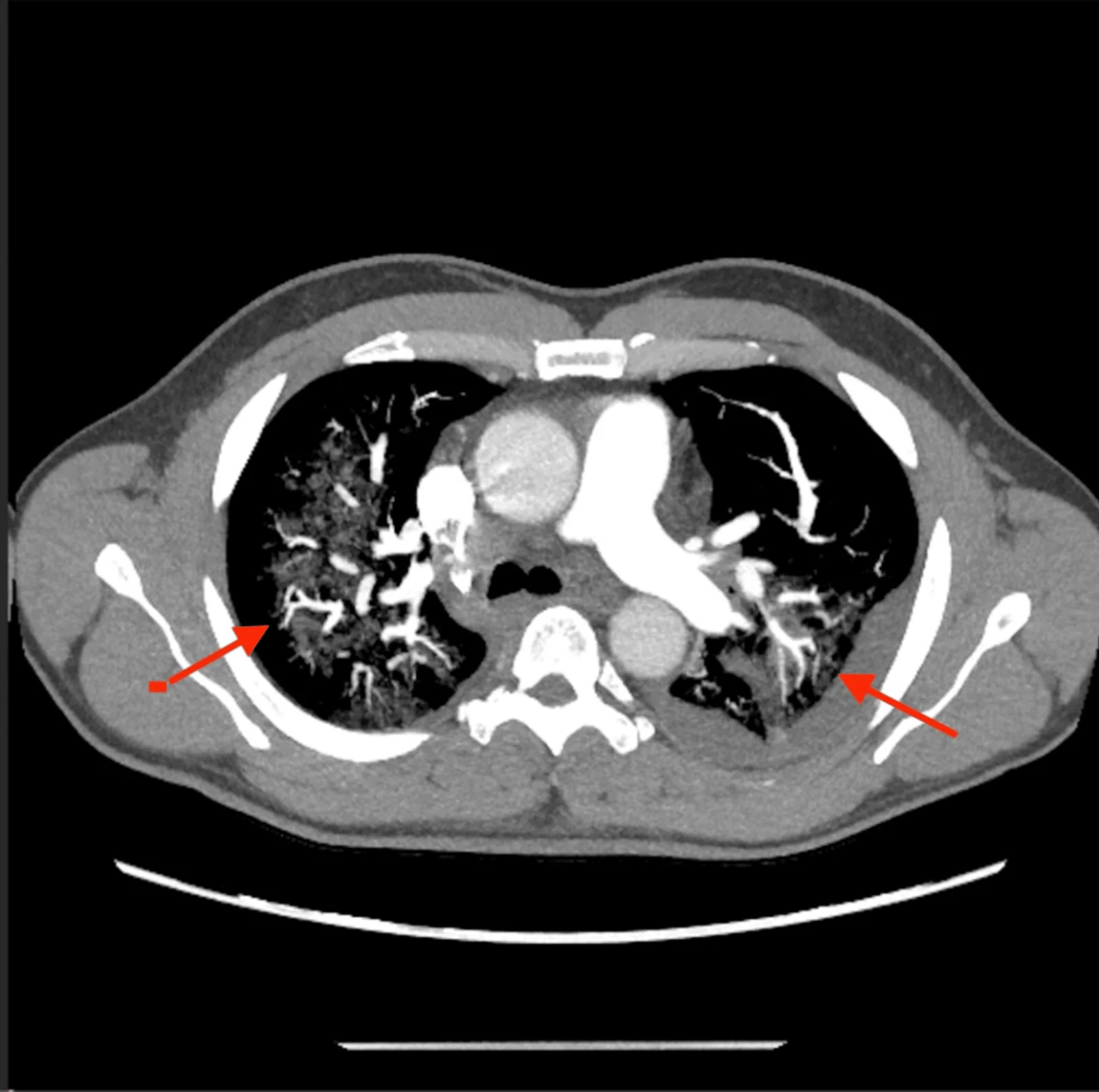
CT chest pulmonary angiogram Bilateral perihilar ground-glass opacities and interlobular septal thickening are observed (red arrows).

Due to concern for community-acquired pneumonia, the patient was started on ceftriaxone and azithromycin, and IV fluids. CT pulmonary angiogram was negative, but CT imaging (Figure 1) revealed bilateral perihilar ground‑glass opacities with interlobular septal thickening, a pattern characteristic of "crazy‑paving.” Chest radiograph showed left perihilar opacity and pleural effusion. Empiric antibiotics remained ineffective in alleviating symptoms.

On the second day of admission, an infectious etiology workup was conducted; however, the *Legionella* urinary antigen test, respiratory viral panel, and methicillin-resistant *Staphylococcus aureus* (MRSA) nares test were negative. Blood and urine cultures were negative the following day. On the third day of hospitalization, after unsuccessfully weaning the patient off the nasal cannula, the patient was switched to a high-flow nasal cannula. On the fifth day of hospital admission, serologic testing confirmed murine typhus IgG antibody levels (1:1024). The patient was started on a 14-day course of 100 mg doxycycline twice daily. After one day, the patient was weaned off oxygen completely and passed the six-minute walk test. He was discharged and instructed to finish the full 14-day course. Four-week follow-up imaging showed near-complete resolution of the ground-glass opacities with interlobular septal thickening.

## Discussion

This case broadens the pulmonary spectrum of murine typhus. While *R. typhi* has been documented to cause cases of pulmonary infiltrates, acute respiratory distress syndrome, pulmonary embolism, and respiratory failure [2], this case presents as a unique instance of an *R. typhi* infection resembling pulmonary alveolar proteinosis. ​​​​​*R. typhi* infects vascular endothelium, leading to systemic inflammation, capillary leak, and noncardiogenic pulmonary edema. These inflammatory changes may transiently impair alveolar macrophage function and surfactant clearance, producing a “crazy-paving” pattern classically associated with PAP. Although idiopathic PAP results from granulocyte-macrophage colony-stimulating factor (GM-CSF) autoantibodies, secondary forms may emerge from transient macrophage suppression due to infection, as may have occurred in this case.

Importantly, the differential diagnosis of crazy-paving opacities besides PAP is broad and includes diffuse alveolar hemorrhage, *Pneumocystis jirovecii* (*P. jirovecii*) pneumonia, cardiogenic and noncardiogenic pulmonary edema, and organizing pneumonia. Diffuse alveolar hemorrhage was considered less likely given the absence of hemoptysis and supportive laboratory evidence. *P. jirovecii* pneumonia was unlikely in the absence of immunosuppression and typical radiographic distribution. Cardiogenic pulmonary edema was not supported by clinical evaluation or cardiac findings. Organizing pneumonia was also considered less consistent given the acute onset of symptoms and rapid response to doxycycline therapy. In this case, systemic symptoms, including thrombocytopenia and multi-organ involvement, pointed towards an acute infectious cause rather than an autoimmune etiology. The absence of anti-GM-CSF antibodies or underlying malignancy further favored a secondary cause.

Prompt initiation of doxycycline is crucial in suspected rickettsial infections. Delays in doxycycline therapy correlate with worsened outcomes, including pulmonary complications and prolonged hospitalization for patients with rickettsial infections [7]. Recent expert reviews emphasize that therapy for sPAP should focus on the underlying cause and that invasive procedures such as bronchoalveolar lavage or biopsy can be deferred in cases with rapid clinical and radiologic improvement [3]. This patient's course underscores the reversibility of infection-associated alveolar macrophage dysfunction with appropriate treatment.

## Conclusions

To our knowledge, this is the first reported case of murine typhus that presents like PAP both on radiological imaging and clinically. Consequently, murine typhus should be considered in those residing in or having traveled to endemic regions who present with diffuse alveolar disease with crazy‑paving patterns. Despite presenting with febrile symptoms, thrombocytopenia, and elevated procalcitonin, those who fail to respond to antibiotics need to be assessed for alternative diagnoses. Depending on the patient's clinical stability and diagnostic uncertainty, early initiation of doxycycline may circumvent the need for invasive diagnostic procedures and can lead to a prompt recovery. This case underscores the importance of interpreting patients' symptoms, laboratory results, and imaging findings within the broader epidemiologic context in which they occur.
